# Fate Mapping of Dendritic Cells

**DOI:** 10.3389/fimmu.2015.00199

**Published:** 2015-05-04

**Authors:** Mateusz Pawel Poltorak, Barbara Ursula Schraml

**Affiliations:** ^1^Institute for Medical Microbiology, Immunology and Hygiene, Technische Universität München, Munich, Germany

**Keywords:** dendritic cell, ontogeny, fate mapping, lineage tracing, mononuclear phagocyte

## Abstract

Dendritic cells (DCs) are a heterogeneous group of mononuclear phagocytes with versatile roles in immunity. They are classified predominantly based on phenotypic and functional properties, namely their stellate morphology, expression of the integrin CD11c, and major histocompatibility class II molecules, as well as their superior capacity to migrate to secondary lymphoid organs and stimulate naïve T cells. However, these attributes are not exclusive to DCs and often change within inflammatory or infectious environments. This led to debates over cell identification and questioned even the mere existence of DCs as distinct leukocyte lineage. Here, we review experimental approaches taken to fate map DCs and discuss how these have shaped our understanding of DC ontogeny and lineage affiliation. Considering the ontogenetic properties of DCs will help to overcome the inherent shortcomings of purely phenotypic- and function-based approaches to cell definition and will yield a more robust way of DC classification.

## Introduction

Dendritic cells (DCs) were originally identified in mouse spleen for their unique stellate morphology, their ability to adhere to certain glass surfaces and their superior capacity to activate naïve T lymphocytes that distinguished them from macrophages (MØs) (1–3). Mostly for historical reasons, DCs are considered part of the mononuclear phagocyte (MP) system, which groups all highly phagocytic cells derived from monocytes or their precursors based on the premise that tissue MØs arise from monocytes (4–9). This presumed relatedness of DCs, monocytes, and MØs coupled to the lack of reliable ways to distinguish MP subtypes has caused continuous debates over accurate cell-type identification and has led some to question whether DCs in fact constitute an independent cell lineage (6, 7, 10–14). However, today we have conclusive evidence demonstrating that DCs, monocytes, and MØs have distinct cellular origin and we further distinguish plasmacytoid DCs (pDCs) from two subsets of so-called conventional or classical DCs (cDCs) based on unique developmental requirements (7, 15–19). Nonetheless, DCs remain defined based on phenotypic and functional properties that often overlap with those of monocytes or MØs (19), although some have suggested a shift in paradigm toward a nomenclature that takes cell ontogeny into account (6, 7, 10).

Dendritic cells are generally identified by their high expression of major histocompatibility complex class II molecules (MHCII) and of the integrin CD11c, as well as their superior capacity to migrate from non-lymphoid to lymphoid organs and stimulate naïve T cells (3, 20–22). However, these characteristics are not absolute and can change in situations of inflammation or infection, thus complicating cell identification (6, 7, 23, 24). For instance, CD11c, considered the hallmark surface marker of DCs, is also found on B, T, and NK cells as well as some monocytes, MØs, and eosinophils (25–32). Dendritic protrusions have also been observed in some MØs and T cells (33–35). Further, surface markers, such as F4/80, CD14, or CD64 (Fc-gamma receptor 1), generally associated with monocytes or MØs can be found on DCs (36–38). One might argue that the most defining feature of DCs is their ability to activate T cells, however such definition discounts the fact that DCs potently regulate innate immune responses independent of their ability to migrate to lymphoid organs or stimulate T cells (39–44). Conversely, non-DCs can carry antigen to lymph nodes and activate naïve T cells in some instances (45–47).

Therefore, morphological and functional properties, as well as the expression of surface markers are insufficient to clearly distinguish DCs from monocytes and MØs, raising the necessity to find a more robust way of cell identification. Recent studies in mouse and human indicate that DCs, MØs, and monocytes have unique ontogenetic properties and thus can be considered distinct cell lineages (36, 48–54). Here, we review approaches that have been employed to track and define the progeny of DC precursors *in vivo* and discuss how such “fate mapping” approaches have improved our understanding of DC heterogeneity and ontogeny. These studies lay the foundation for moving toward cell ontogeny as a major lineage-determining criterion, which will allow for a more reliable and precise classification of DCs and DC subsets.

## DC Development

**Figure 1:**
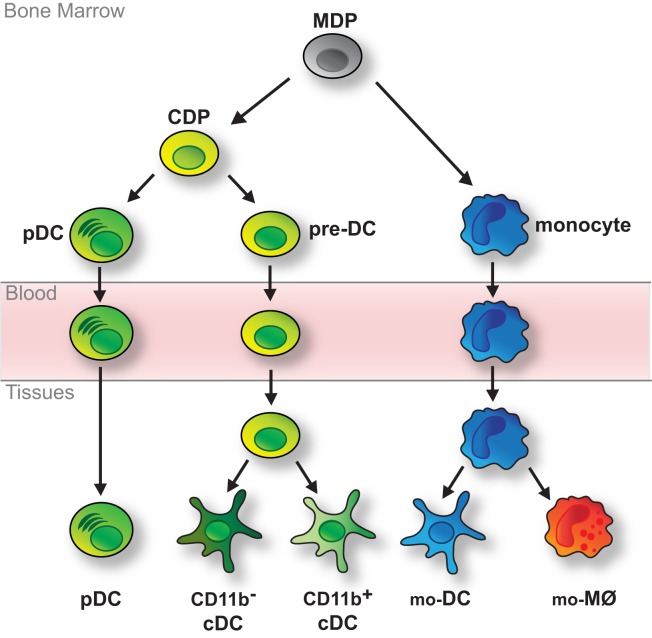
**Classic model of DC development**. DCs and monocytes are ancestrally related and arise from bi-potential MDPs residing in the bone marrow. MDPs further differentiate into monocytes and CDPs, which are restricted to the generation of various types of DCs. CDPs give rise to pDCs, which fully develop in the bone marrow, and pre-DCs, which migrate through the blood to tissues, where they fully differentiate into CD11b^−^ (including CD8α^+^ cDCs in lymphoid tissue and migratory CD103^+^ cDCs in non-lymphoid tissue) and CD11b^+^ cDCs. Monocytes complete their development in the bone marrow and reach peripheral tissues via the bloodstream. There they further differentiate into monocyte-derived DCs (mo-DCs) or MØs (mo-MØs) in response to environmental cues.

Dendritic cells are short-lived and their maintenance relies on constant replenishment from bone marrow progenitors that originate from hematopoietic stem cells (HSCs) (19, 55). In the classic model of DC development monocytes and DCs arise from bi-potent progenitors, so-called MØ and DC progenitors (MDPs) (Figure 1) (56). MDPs further give rise to common DC progenitors (CDPs) restricted to the generation of pDCs and cDCs (Figure 1) (57, 58). pDCs terminally differentiate in the bone marrow, thus exit the bone marrow as fully developed cells and reach peripheral organs via the blood stream (Figure 1) (15, 59). In contrast, cDCs arise from another developmental intermediate termed pre-DC, which exits the bone marrow and migrates through the blood to seed lymphoid and non-lymphoid tissues (60, 61). There, pre-DCs terminally differentiate into cDCs, including the main CD11b^−^ and CD11b^+^ subtypes (Figure 1) (60–63). In lymphoid tissues these are CD8α^+^CD11b^−^ and CD11b^+^ resident cDCs, whereas in non-lymphoid tissues they comprise CD103^+^CD11b^−^ and CD11b^+^ migratory cDCs (3, 60–63). Like pDCs, monocytes complete their development in the bone marrow but in tissues they differentiate into cells with DC- or MØ-like features (Figure 1) (23, 24, 64, 65). This plasticity is remarkably prominent in inflammatory or infectious environments, when monocyte-derived cells with qualities of DCs have been referred to as TNF-α/iNOS-producing DCs (Tip-DCs), monocyte-derived DCs (mo-DCs), and/or inflammatory DCs (23, 24, 64, 65).

Although most of our knowledge concerning DC development is derived from mouse studies, developmental parallels have been observed in other species (66–73). Especially the identification of putative equivalent DC progenitor populations in human holds promise for future research (72, 73). Yet, some uncertainties remain. Common lymphoid progenitors (CLPs) can give rise to DC descendants upon adoptive transfer (74), although it is now thought that DCs originate predominantly from myeloid progenitors (75, 76). Nonetheless, some pDCs, but not cDCs, show evidence of VDJ gene rearrangements, potentially indicating lymphoid lineage heritage (15, 59, 77). However, it remains unclear whether evidence of *Rag* gene expression history necessarily means that pDCs have dual lymphoid and myeloid origin. Contrary to the dogma that monocytes and DCs share a common immediate ancestor, recent data suggest that lineage divergence of HSC-derived myeloid cells occurs much earlier than previously predicted and that monocytes and DCs might arise independent of a bi-potential developmental intermediate (49, 78, 79). Elucidating such unresolved aspects pertaining to DC ontogeny may solve uncertainties in determining lineage affiliation, which, in turn, will aid to further decipher the unique functions of DCs in immunity.

## Fate Mapping

Understanding cell development requires models with which the relationship of a precursor cell and its progeny can be defined *in vivo*. Such “fate mapping” can be achieved in various ways and relies on the selective labeling of the cell(s) of interest so that consequently the development of the marked cell can be followed in its natural environment (80). Tracing progenitors *in vivo* also offers the possibility to determine the fate of populations when lineage affiliation is most heavily debated, namely following experimental manipulation to generate conditions of inflammation or infection. While most fate mapping strategies follow the progeny of bulk cell populations, recently developed techniques have enabled the tracing of single cells, thus providing valuable information regarding their developmental potential at the clonal level (80, 81). In all fate mapping experiments, it is important to consider that their interpretation is dependent on the use of select, faithful and stable markers (82).

### Precursor transfers

**Figure 2:**
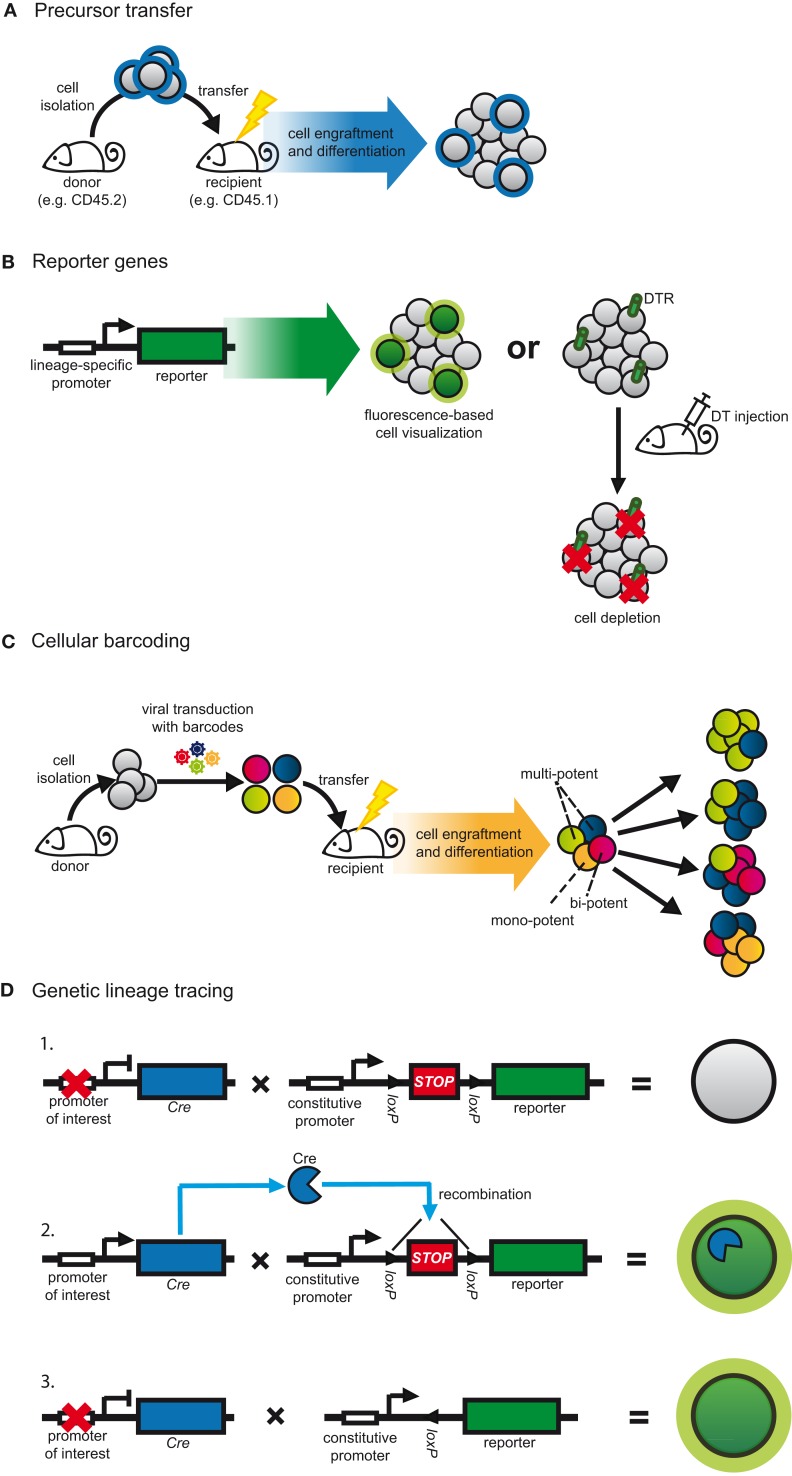
**Strategies to fate map DCs**. **(A)** Progenitors are adoptively transferred to assess their differentiation in the physiological context. Graft-derived cells are distinguished from host cells based on pre-defined labels, for instance congenic markers. This method is often combined with strategies to increase the niche available for cell engraftment, such as irradiation. **(B)** In transgenic approaches, lineage-restricted promoters can be used to drive a reporter gene. Target cell populations can be visualized by the expression of fluorescent proteins or can be depleted. In the latter case, cell-restricted expression of DTR allows for conditional cell ablation following DT injection. **(C)** Progenitors are transduced *in vitro* with semi-random DNA sequences (barcodes) by retro- or lentiviral vectors and subsequently transferred into irradiated congenic recipients. After differentiation, cell progeny are analyzed for their barcode repertoire using deep sequencing or microarray. The representation of a given barcode in multiple cell populations indicates multi-potency of the transferred cell. **(D)** Expression of Cre recombinase is driven by a lineage or cell-specific promoter. Additionally, a reporter gene, usually a fluorescent protein, is placed under control of a constitutive promoter. Expression of the reporter is blocked by inserting a *loxP*-flanked *STOP* cassette (1). Cre expression leads to excision of the *STOP* cassette resulting in expression of the reporter gene (2). Since the promoter-driving reporter gene expression is constitutively active, the target cell is irreversibly labeled irrespective of continuous Cre expression (3).

The transfer of purified and pre-marked precursor cells into congenic recipients is the most accessible form of fate mapping as a variety of labeling options can be used to distinguish between donor and host cells (Figure 2A) (80). As a result, precursor transfers are commonly used to study cell development and lineage relationships and remain a standard protocol for defining the stemness of progenitor cells (80). Such experiments rely on the ability to purify sufficient precursors that, after cell isolation, retain the capacity to home to the appropriate anatomical niche and expand sufficiently into detectable progeny. To circumvent such limitations transfer studies are often combined with protocols to induce leukopenia, such as irradiation, in order to increase the niche available for cell engraftment (Figure 2A) (80). However, these manipulations can alter developmental signals, which, in turn, might impact on the interpretation of results (18, 54, 83). To best mimic the endogenous cellular environment, progenitors have been returned directly to their organs of origin, for instance by intra-bone injection (84).

The DC progenitors MDP, CDP, and pre-DC were in part defined by assessing their developmental potential after adoptive transfer into mice (56–58, 60, 61, 84–86). In such experiments, MDPs give rise to DCs and monocytes, whereas CDPs and pre-DCs are restricted to the generation of DCs but do not generate monocytes or other leukocyte lineages (56–58, 61, 84–86). In combination with experiments assessing the differentiation potential of single progenitors *in vitro* (56–58), these studies have significantly shaped our view of DC development (Figure 1). Surprisingly, the existence of MDP as a bi-potential intermediate for DCs and monocytes has recently been questioned when single CX_3_CR1^+^ MDPs were unable to generate both DCs and monocytes upon differentiation *in vitro* (78). The authors further found that adoptively transferred CX_3_CR1^+^ MDPs, not only gave rise to DCs and monocytes but also neutrophils (78). However, such multi-potency of MDPs was not observed in earlier studies (52, 56, 61, 85, 86) and is not evident in genetic CX_3_CR1 fate mapping experiments (50). It is possible that these discrepancies may be explained by experimental variation such as differences in cell isolation, the timing of analysis or variances in the niche available for cell engraftment following irradiation (18, 54, 83). In light of these results it is noteworthy, however, that upon adoptive transfer MDPs exhibit pDC potential only in some studies (52, 86) but not others (56, 85), whereas the presumed downstream CDPs produce both pDCs and cDCs (57, 58). Taken together these experiments raise some doubt about the existence of a MDP as a key developmental intermediate for monocytes, cDCs, and pDCs. However, resolving this matter will require the use of better models to trace single cells *in vivo* as experiments relying on the isolation and analysis of bulk progenitor populations are inherently prone to disparities in gating strategy or cell purity.

In DC ontogeny, these issues are augmented because MDP and CDP exhibit substantial phenotypic overlap: both lack lineage-defining markers, are characterized by expression of CX_3_CR1, CD115 (M-CSFR, Csf1R) as well as CD135 (FMS-like tyrosine kinase 3, FLT3) and, until recently, CDP could only be distinguished from MDP by lower expression of the receptor tyrosine kinase CD117 (c-kit) (56–58, 61, 86). We have recently found that the C-type lectin receptor DNGR-1 (Clec9a) marks cells resembling CDPs (36). Surprisingly, upon adoptive transfer, DNGR-1^+^CD115^+^ progenitors exhibit cDC-restricted differentiation potential and do not generate pDCs (36), suggesting that DNGR-1 marks cDC-restricted progenitors. These data are in line with a recent study demonstrating a strong bias for CD115^+^ CDPs to generate cDCs, whereas pDCs arise predominantly from CD115 negative cells (79). Therefore, cDCs and pDCs appear to have distinct developmental intermediates that can be distinguished by expression of CD115 (79) and DNGR-1 (36). Since CD115^+^ CDPs presumably express DNGR-1 (36), it is unclear why some CD115^+^ CDPs show combined cDC and pDC potential in clonal assays (57, 58, 79). It is possible that antibody-mediated triggering of DNGR-1 or growth factor receptors, such as CD115, during cell isolation skews DC differentiation toward a particular DC sub-lineage in an unforeseeable manner. The developmental potential of progenitors may also be influenced by the specific culture conditions used (78) or DCs could exhibit a degree of developmental plasticity (87). Nonetheless, the existence of a putative intermediate monocyte-restricted progenitor downstream of MDP (common monocyte progenitor, cMoP) (52) alongside the aforementioned pDC- and cDC-restricted progenitors supports a model in which monocytes, cDCs, and pDCs develop independently. The genuine point of lineage divergence, however, remains to be determined.

Questions regarding the lineage affiliation of DCs have been muddled significantly by the developmental plasticity of monocytes (6, 24). The phenotypic transformation of monocytes into DC-like cells is most prominent in inflamed environments (8, 19, 23, 24). It can also be mimicked *in vitro* by culturing monocytes in the presence of GM-CSF (granulocyte-macrophage colony-stimulating factor) ± IL-4 (Interleukin-4) (88, 89). However, *in vivo* the inflammation-induced differentiation of monocytes into cells with attributes of DCs appears GM-CSF-independent (90), highlighting that the developmental requirements underlying this phenotypic conversion *in vitro* might differ from those involved *in vivo*. In the absence of experimentally induced infection or inflammation, adoptively transferred monocytes readily acquire CD11c and MHCII expression as well as functional features of DCs in non-lymphoid tissues (91–95). This phenotypic conversion is also observed after adoptive transfer into unirradiated hosts, which most closely mimics steady-state conditions (63). In contrast, transferred monocytes do not generate DCs in lymphoid organs, even if the niche for engraftment is opened by depletion of CD11c^+^ cells (84). Importantly, in non-lymphoid tissues monocytes exclusively generate CD11b^+^, but not CD103^+^CD11b^−^ cells, which is in contrast to CDPs and pre-DCs that generate CD11b^+^ as well as CD103^+^CD11b^−^ cDCs (63, 91–95). Therefore, CD11c^+^MHCII^+^CD11b^+^ cells in non-lymphoid tissues appear to constitute a population of mixed cellular origin that can arise from monocytic progenitors as well as pre-DCs. Adoptive transfer experiments do not allow to determine the relative contribution of each progenitor to this population, although surrogate markers such as CD64 or Mar-1 can serve to distinguish monocyte-derived cells from bona fide pre-DC-derived cDCs (46, 93, 94).

Notably, in irradiated hosts transferred monocytes can also generate CD11c^+^MHCII^+^ cells of the epidermis, which resemble Langerhans cells (LCs) (96–99). LCs exhibit many phenotypic and functional features of DCs, such as the capacity to migrate to lymphoid organs and stimulate naïve T cells, and have long been considered a prototypical DC population (96–99). However, we now realize that the majority of LCs is established before birth and maintained under steady-state conditions by self-renewal from local progenitors (96, 97, 99–102). These properties thus ontogenetically separate LCs from bone marrow-derived DCs or monocyte-derived cells. Moreover, monocytes may not necessarily adopt features of DCs or MØs upon entry into tissues, as a recent study indicates that monocyte can also exist in tissues without further differentiation (45). When considering this immense plasticity it will be crucial to elucidate the environmental cues that shape the diverse fates of monocytes to further dissect the full functional spectrum of monocytes and monocyte-derived cells.

### Lineage restricted reporters

When the availability of isolatable progenitor cells is limiting and when populations are ontogenetically heterogeneous or might be influenced by alterations in their surroundings, determining lineage affiliation requires models to trace cells directly in their natural environment. One way to achieve this is by engineering models in which lineage-restricted promoters or genetic elements drive the expression of reporter genes (Figure 2B) (80, 82, 103). It is important to bear in mind that such experiments assume that the expression of the selected marker is restricted to the cell lineage in question and therefore, the choice of stable and specific markers is essential (80, 82, 103). Additionally, the genetic elements used to drive expression of the reporter must faithfully mimic endogenous gene expression (80, 82, 103).

Genetic elements of the *Itgax* gene, which encodes CD11c, have extensively been used to generate reporters to study DCs (82, 103). As such, transgenic mice in which the CD11c promoter drives the expression of fluorescent proteins (Figure 2B) have been key to visualizing the distribution and cellular interactions of DCs in a variety of tissues, including lymphoid organs, heart, lung, and skin (103–107). But fate mapping can also be achieved by cell deletion. Transgenic expression of primate diphtheria toxin receptor (DTR) renders murine cells susceptible to diphtheria toxin (DT)-induced cell death and, thus, enables inducible target cell depletion (Figure 2B) (82, 108). In this sense mice in which DTR expression is controlled by the elements of the CD11c promoter have been widely used to characterize the *in vivo* functions of DCs (28, 109–112). In part through analyzing such reporter mice, however, it has become evident that CD11c expression is not entirely restricted to DCs. It is also expressed on alveolar MØs, Ly6C^low^ as well as activated monocytes, plasmablasts, NK cells, and some T cells (25–29, 113). In addition, CD11c-driven fate reporter expression varies depending on the specific promoter elements used for transgenesis. CD11c.DTR mice, which were generated by conventional transgenesis using a 5.5-kb promoter element of the *Itgax* gene (109, 114), efficiently deplete most CD11c-expressing cDCs, LCs, alveolar, splenic marginal zone, and metallophilic MØs, as well as plasmablasts and T cells (27, 109, 115). However, DT-induced cell depletion in these mice is incomplete and spares certain cell types that transcribe their endogenous *Itgax* allele, including pDCs and NK cells (82, 115). Additionally, prolonged cell depletion using CD11c.DTR mice requires the use of bone marrow chimeras, possibly because of aberrant DTR expression on non-immune cells (82, 108, 112). Notably, this is not the case in CD11c.DOG and CD11c.LuciDTR mice, which were generated using bacterial artificial chromosome (BAC) transgenesis to place DTR under control of the extended regulatory region of the *Itgax* gene and in which DTR expression seems to more faithfully represent endogenous CD11c expression (28, 112, 115, 116). In all models, the occurrence of systemic neutrophilia and monocytosis following CD11c^+^ cell depletion (28, 115, 117) adds another layer of complexity to deciphering the cellular function and lineage affiliation of DCs.

The realization that CD11c is not restricted to DCs in all instances nurtured the search for more specific lineage-defining markers. Two groups simultaneously identified the transcription factor Zbtb46 (zDC, Btbd4) as ideal candidate to distinguish cDCs, as it is expressed in pre-DCs and cDCs but not in pDCs or their precursors (37, 38). Consistently, CD8α^+^ and CD11b^+^ cDCs in lymphoid organs as well as CD103^+^ cDCs in non-lymphoid organs uniformly express Zbtb46 as assessed in Zbtb46-GFP (37) and Zbtb46-DTR (38) reporter mice generated by site-directed mutagenesis. In contrast, CD11c^+^MHCII^+^CD11b^+^ cells in non-lymphoid organs, including lung, small intestine, and kidney, exhibit partial Zbtb46 expression (37, 38) indicating that they represent a heterogeneous population. This is consistent with reports demonstrating that these cells are of mixed monocyte and pre-DC origin (63, 91, 92, 95). Subsequently, Zbtb46 reporter mice have been used to help establish lineage relationships in a variety of tissues including heart, pancreas, tumors, and thymus (118–121). The fact that Zbtb46 expression is also found in human DCs suggests that it may also help to identify DCs across species (48, 122).

Nevertheless, the use of Zbtb46 as lineage-defining marker requires a note of caution. Zbtb46 expression is downregulated after DC stimulation and it is found in some non-immune cells (37, 123). Despite its prominent expression in the cDC lineage, Zbtb46 appears largely dispensable for cDC development (37, 123). Instead, it may reinforce DC-specific transcriptional programs (37) and/or suppress DC activation (123). Interestingly, monocytes activated in the presence of GM-CSF ± IL-4 uniformly induce Zbtb46 expression, whereas monocyte-derived Tip-DCs that are generated following infection with *Listeria monocytogenes* do not (37). This raises the possibility that Zbtb46 may control DC-like features of monocyte-derived cells in some inflammatory situations and it will be interesting to determine if Zbtb46 controls transcriptional programs in monocytes. These data also highlight that despite its selective expression on cDC progenitors and their descendants, Zbtb46 is not necessarily an indicator of cell ontogeny.

### Identifying common developmental requirements

Establishing that the development and/or delineation of a cell type depends on a certain transcription or growth factor constitutes a powerful way of fate mapping that has extensively been applied to MPs (42, 51, 63, 124–141). We can now clearly delineate DCs into distinct subpopulations based on the transcriptional programs that govern their development. pDCs are distinguished from two subsets of cDCs by their dependence on E2-2 (67, 142). The differentiation of pre-DCs into CD8α^+^ cDCs in lymphoid organs and CD103^+^CD11b^−^ cDCs in non-lymphoid tissues is controlled by a set of transcription factors, including Irf8, Nfil-3, Id2, and Batf3 (124–128). Therefore, CD8α^+^ cDCs and CD103^+^ cDCs represent a developmentally related lineage of cDCs (6, 7). Notably, these cells also exhibit a degree of functional relatedness that is, for instance, exemplified by their superior capacity to activate CD8^+^ T cells (124, 143–145). In contrast, the development of CD11b^+^ cDCs from pre-DCs is controlled by distinct transcription factors, including RelB, RbpJ, PU.1, and Irf4 (42, 129–136). Notably, expression of CD24 separates pre-DCs into cells that preferentially generate either CD8α^+^ or CD11b^+^ cDCs in spleen (60) suggesting a stepwise differentiation of pre-DCs into cDCs. It will be interesting to determine whether such heterogeneity of pre-DCs also exists in the bone marrow. Notably, the extent of transcription factor dependence is linked to the genetic background of the particular mouse strain analyzed (146–148), indicating that transcriptional requirements are not always absolute or redundant factors exist (148). Consistently, CD8α^+^ DCs can develop in the absence of Batf3, Id2, and Nfil-3 (149). The local microenvironment may also contribute to shaping the diversity of the DC compartment, as in some tissues, such as the spleen and intestinal system, CD11b^+^ cDCs can be divided into ontogenetically and functionally distinct subpopulations (36, 42, 91, 95, 131). Importantly, some of the transcription factors controlling DC differentiation in mice have also been implicated in the development of human DCs (67, 69, 71) and putative equivalent DC subpopulations exist in rat, chicken, sheep, and pig (150–153), highlighting that DC populations are conserved across species.

While several growth factors have been linked to DC differentiation, the development of all DC subsets is strongly dependent on FLT3 ligand (FLT3L) and downstream signaling events (7, 18, 154). FLT3L administration potently expands pDCs and cDCs in mice and humans (72, 73, 85, 155–157). *In vitro*, FLT3L promotes the differentiation of bone marrow progenitors from mice, humans, and pigs into functional subsets of DCs (66, 158, 159). Mice lacking FLT3L display a severe deficiency in DCs, which is also apparent, although to a lesser extent, in mice lacking its receptor CD135 or mice treated with CD135 inhibitors (63, 137, 160, 161). In contrast, FLT3L appears largely dispensable for monocyte and MØ development (137) and, therefore, FLT3L dependency is often used delineate DCs *in vivo* (18, 65, 162). The interpretation of fate mapping using mice deficient in CD135 or its ligand is however complicated by the fact that these animals also exhibit abnormalities in other hematopoietic lineages, including B, T, and NK cells (137, 163) and show evidence of systemic neutrophilia and monocytosis, as has been reported in other DC-deficient models (112, 117).

Despite the prominent expression of CD135 on DC progenitors it remains to be clarified exactly at what stage of cellular differentiation FLT3L impacts on DC development. Consistent with a role for FLT3L early in development, a reduction of bone marrow CDPs in FLT3L deficient animals has been reported but ranges from a mere twofold decrease (164) to near complete absence (78). In contrast, the numbers of MDPs and splenic pre-DCs appear largely unaffected by CD135 deficiency (85). The observation that pre-DC frequencies in non-lymphoid organs of FLT3L-deficient mice are reduced (63) and that transfer of DCs into a FLT3L-deficient environment decreases their homeostatic proliferation (85) indicates a role for FLT3L in the peripheral expansion of DCs rather than their differentiation. This interpretation would equally be consistent with the observation that DCs that develop in the absence of FLT3L are functional (137). In light of this finding it will be interesting to determine, to what extent FLT3L impacts on the development and functional regulation of other MPs. Addition of FLT3L to purified human monocytes cultured with GM-CSF ± IL-4 increases their T cell stimulatory capacity (165), although it is not clear whether this is also the case for murine monocytes. Culture of murine bone marrow with GM-CSF and IL-4 presumably mimics monocyte differentiation under the same conditions (166). When FLT3 signaling is inhibited in such bulk cultures the T cell stimulatory capacity of the output cells is reduced (161). Therefore, these data raise the possibility that FLT3L might influence monocyte differentiation into cells with functional properties of DCs also in the murine system, although a direct causality remains to be demonstrated. Further, comparative gene expression profiling revealed that upon migration to lymph nodes LCs induce CD135 expression (167), indicating that they might be capable of responding to FLT3L. Therefore, it is conceivable that FLT3L may control certain functional aspects generally associated with DCs, such as antigen presentation, in ontogenetically distinct MP subtypes, which will be interesting to formally address in the context of FLT3L or CD135 deficiency.

Dendritic cell progenitors also express CD115, the receptor for MØ colony-stimulating factor (M-CSF) (56–58, 61, 86). However, compared to the dominant role of FLT3L in DC differentiation, M-CSF-deficiency only mildly impacts on DC development (168). M-CSF deficient osteopetrotic (op/op) mice exhibit a two- to threefold reduction in splenic cDCs and pDCs, respectively, but the remaining DCs are capable of stimulating a mixed lymphocyte reaction and induce costimulatory molecules upon activation, thus appear functional (168). In contrast, M-CSF is strongly required for monocyte and MØ development (141, 169). Therefore, the observation that mice lacking CD115 exhibit reduced frequencies of CD11c^+^MHCII^+^CD11b^+^ cells in non-lymphoid organs (63, 91) likely reflects the ontogenetic heterogeneity of this population (63, 91–95). Consistently, M-CSF is also required for the generation of monocyte-derived cells with features of DCs during inflammation (90). Nonetheless, M-CSF may play a role in DC development. It can promote DC differentiation *in vitro* and *in vivo* even in the absence of FLT3L, although DCs generated by M-CSF alone phenotypically and functionally differ from those induced by FLT3L (170). M-CSF-induced DC poeisis is also more efficient in FLT3L-sufficient conditions (170). *In vivo*, antibody-mediated blockade of M-CSF in pregnant mice reduces pre-DC extravasation, translating into a reduction of CD11b^+^ DCs in the pregnant uterus (171). Whether M-CSF affects pre-DC migration also in other tissues and whether it acts in a cell intrinsic manner or by promoting the production of chemotactic factors by other cells remains to be determined (171).

In purified monocytes, GM-CSF induces phenotypic and functional attributes of DCs (88, 89, 172). Similarly, purified CD115^+^ MDPs respond to GM-CSF by differentiating into CD11c^+^MHCII^+^ DCs (85) and GM-CSF deficiency leads to a slight reduction of bone marrow MDPs and CDPs (164). However, GM-CSF is dispensable for the differentiation of lymphoid tissue DCs (85, 173) and, therefore, it seemed likely that GM-CSF would selectively regulate the differentiation of monocytes into cells resembling DCs (23). This speculation also lead to the hypothesis that monocytes cultured in the presence of GM-CSF represent the counterpart of mo-DCs generated under conditions of inflammation/infection *in vivo* (23). Surprisingly, GM-CSF does not appear to control monocyte differentiation *in vivo* (90) and thus, GM-CSF elicited monocyte-derived cells are unlikely to be fully equivalent to inflammatory monocyte-derived cells. Rather, GM-CSF influences the homeostasis of cDCs in a variety, but not all, non-lymphoid tissues, most likely by promoting cell survival (90). Importantly, GM-CSF deficiency leads to a greater reduction of CD103^+^ cDCs than of CD11b^+^ cDCs (90). However, the extent of cDC reduction in the absence of GM-CSF apparently relates to the markers used for cell identification (90, 147, 164). This is most likely because GM-CSF regulates certain phenotypic as well as functional features of DCs, such as CD103 expression (174) or their ability to cross-present antigen (90, 174, 175). Therefore, the above-mentioned growth factors not only influence lineage decisions but also impact on the functional regulation of DCs, monocytes, and MØs. Elucidating the exact roles of FLT3L, GM-CSF, and M-CSF in each cell type will help to decipher the functional heterogeneity of MPs.

### Cellular barcoding

The biggest challenge for fate mapping is to trace the developmental plasticity of individual cells. This can now be achieved using “cellular barcoding,” in which progenitors are tagged *in vitro* with semi-random, non-coding DNA sequences by transduction using retro- or lentiviral vectors (Figure 2C) (81). Therefore, the barcodes are heritable and by choosing conditions of low transduction efficiency one can ensure that each cell receives only a single barcode. Subsequently, barcode-labeled progenitors are adoptively transferred in numbers low enough to minimize the chance that two identically barcoded cells are transferred into the same recipient (Figure 2C). After differentiation *in vivo*, cell progeny are analyzed for their barcode repertoire using deep sequencing or custom microarray. Since each barcode represents an individual progenitor, the presence of the same barcode in more than one cell type indicates that they were generated from a single precursor (multi-potent or bi-potent, Figure 2C). On the other hand, if a barcode is only found in one cell type, the progenitor generated only a single cell lineage (mono-potent, Figure 2C) (81).

During maturation, HSCs are thought to progressively lose their self-renewal ability and become increasingly limited in their differentiation potential, ultimately giving rise to lineage-restricted progenitors (55, 176). Lymphoid primed multi-potent progenitors (LMPPs) are developmental intermediates downstream of HSCs that can give rise to various, but not all, cell lineages and are thus considered multi-potent (55, 176). Surprisingly, in barcoding experiments only a minority (3%) of single LMPPs exhibits true multi-potency, defined as the ability to generate all of the following cell lineages: B cells, DCs, and myeloid cells (monocytes and neutrophils) (49). Rather, single LMPPs differ drastically in terms of their cellular output: 10% of the progenitors contribute primarily to B cells, 10% primarily to myeloid cells but about 50% of transferred LMPPs produce predominantly DCs (49). The remaining fraction of progenitors exhibits bi-potentiality to generate combinations of the examined cell lineages (49). Therefore, LMPPs are multi-potent when analyzed as a population, however single cells exhibit unexpected lineage bias that is imprinted early in development. Why the majority of LMPPs is DC-committed (49), even though DCs constitute a minority lineage compared to B cells, remains to be clarified, although it is possible that some progenitors proliferate better than others or have certain competitive advantages. A major lineage divergence toward DCs seems to occur before or at the LMPP stage, as most HSCs analyzed by the same method are multi-potent, although even HSCs exhibit a degree of lineage bias (49, 177). Since CDPs might arise directly from LMPPs without additional developmental intermediates (79), these data infer that DCs diverge as a developmental lineage distinct from other myeloid cells early on (49).

This, again, questions the existence of a bi-potential MDP as central intermediate in the development of DCs and monocytes. Yet, it is noteworthy that even though DC-biased LMPPs are fivefold more frequent than bi-potent myeloid/DC LMPPs, mono-potent and bi-potent progenitors contribute equally to the final DC pool (49). Therefore, bi-potent progenitors seem to play a significant part in generating DCs, potentially because they have a proliferative advantage. Resolving these issues will require further refinement of the technique at hand. The differentiation potential of progenitors may be influenced by cell isolation, processing or *in vitro* manipulation (80) and virus-mediated transformation might skew cell fate in an unforeseeable manner, as evidenced by the fact that barcoded LMPPs cannot generate T cells (49, 81). This also means that barcoding does not yet uncover the full potential of single progenitors. The early lineage bias of HSCs and LMPPs suggests that cell development may follow a model of graded commitment rather than proceeding in a truly stepwise manner (178). It will be interesting to determine, to what extent this process is regulated by epigenetic modification and how inflammatory processes might impact on lineage divergence. Future studies will benefit from the development of models allowing for *in vivo* barcoding of single cells but the labor-intensive quantification and analysis of barcoding experiments makes it difficult to follow populations in real time.

### Genetic lineage tracing

Dynamic mapping of populations of distinct origin *in vivo* can be achieved using genetic lineage tracing based on *Cre-loxP* technology (Figure 2D) (80, 179). It relies on inducible reporter genes that are placed under the control of constitutively active promoters, such as the *Rosa26* locus. The reporter is most commonly a fluorescent protein that is preceded by a *loxP-*flanked *STOP* cassette and, therefore, its expression is induced only after Cre recombinase (Cre) mediated excision of the stop codon (Figure 2D). Since this form of labeling is genetic it is also heritable, meaning that any cell expressing Cre will pass on the label to all progeny, irrespective of continuous recombinase expression (Figure 2D). Since the promoter driving the reporter gene is constitutively active, labeling is irreversible and not affected by fluctuations in gene expression (Figure 2D) (80).

By crossing mice expressing Cre under the control of the *Clec9a* locus to *Rosa26*-*STOP-flox*-enhanced-yellow fluorescent protein (YFP) reporter mice (180), we have recently generated the first genetic model to trace the progeny of DNGR-1^+^ CDPs and pre-DCs (36). In these mice, YFP expression is restricted to DCs but is not found in monocytes or MØs even in inflammatory conditions, as tested after intestinal inflammation or infection with *L. monocytogenes* (36). Nonetheless, certain limitations need to be taken into account. DNGR-1 is also expressed on CD8α^+^/CD103^+^ cDCs and to a lower extent on pDCs (36, 71, 181, 182) and, therefore, in these populations labeling is not a strict indicator of cell ontogeny. Further, labeling of CDP and pre-DC progeny in mice heterozygous for Cre is incomplete, possibly due to a delay in Cre protein synthesis and DNA recombination in rapidly cycling progenitors (36). Consistently, penetrance of the YFP label is increased in mice homozygous for Cre (36). The efficiency of lineage tracing experiments in such cases or when Cre expression is low may be improved by using alternate reporter constructs in which the *loxP* sites are positioned closer together, thus facilitating recombination (183).

Genetic lineage tracing does not require prior knowledge of which markers are expressed by the output cells and, thus, enables unbiased monitoring of cell ontogeny. Therefore, we were able to identify CDP-derived cells in cell populations previously thought to constitute monocytes/MØs based on the expression of surface markers, such as CD64 (36). CD64^+^ CDP-derived cells do not express *Clec9a* message and are especially frequent in kidneys, although the presence of few YFP^+^ cells in the CD64^+^ component of lung and small intestine indicates that atypical CDP-derived cells also exist in other tissues (36). CD64^+^ kidney DCs resemble yolk sac-derived F4/80^hi^ tissue-resident MØs, appear to lack Zbtb46 expression (37) and their affiliation as DCs or MØs has been debated (184). We, therefore, used adoptive transfer as additional method to confirm cell ontogeny. Surprisingly, neither purified DNGR-1^+^ CDPs nor total bone marrow generated F4/80^hi^CD64^+^ CDP progeny in kidneys 1 week after adoptive transfer into irradiated recipients (36). Since kidney DCs reportedly have a slow turnover (185), it is possible that CDPs had insufficient opportunity to reach their renal niche and expand during short-term transfer experiments. Consistent with this notion, F4/80^hi^CD64^+^ kidney leukocytes were efficiently generated from bone marrow progenitors in long-term reconstitution experiments (36). Therefore, our data strongly support a CDP origin of CD64^+^ kidney leukocytes, despite their phenotypic resemblance to monocytes or MØs (36). These data exemplify the power of lineage tracing in following cell ontogeny in an unbiased way, although it is possible that DNGR-1 is expressed on yet unidentified developmental intermediates.

Addressing this possibility might require tamoxifen-inducible Cre constructs that can be used to pulse label progenitor populations (80). In the future, combinatorial approaches, such as “split-Cre” fragments controlled by two different promoters (186) or an intersection where Cre and the inducible reporter are driven by two cell-specific promoters (187, 188) may be of benefit to generate improved models to lineage trace DCs. The identification of CDP-derived cells with attributes of monocytes/MØs exemplifies the insufficiency of phenotypic properties, such as surface markers, as means of accurate cell identification of MPs. It also raises the question why cells of distinct ontogeny but overlapping phenotype exist in the same tissue. Further elucidation of the specific functions of MPs in immunity will benefit from lineage tracing approaches that result in target cell deletion through the use of inducible DTR or DT subunit modules (82, 112, 189, 190).

## Conclusion

**Figure 3:**
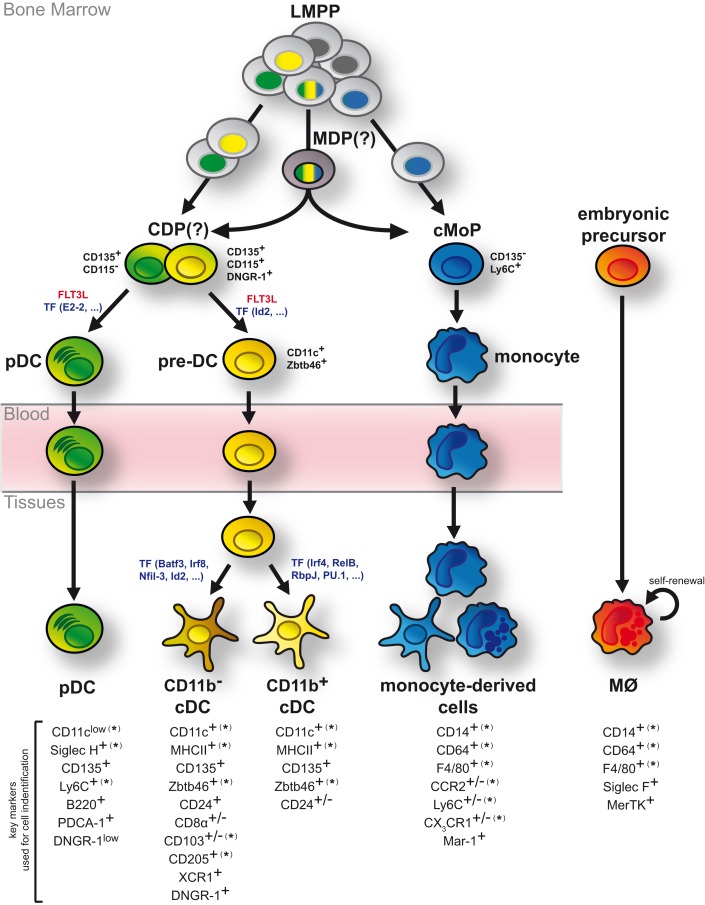
**DCs develop as independent cell lineage**. Although bone marrow LMPPs are generally considered multi-potent, single LMPPs exhibit a degree of lineage bias toward generating exclusively DCs or monocytes. Such mono-potent LMPPs (single colored nuclei) may generate DC progenitors and monocyte progenitors (cMoPs) directly without additional developmental intermediates. In contrast, multi-potent LMPPs (tri-colored nuclei) presumably give rise to DCs and monocytes via bi-potent MDPs and CDPs. CDPs separate into pDC- and cDC-biased DC progenitors and can be delineated from cMoPs based on the expression of surface markers, including CD135, DNGR-1, Ly6C, and CD115 (as indicated in black). While pDCs fully develop in the bone marrow, cDCs arise via pre-DCs, which migrate through the blood stream to lymphoid and non-lymphoid tissues, where they terminally differentiate into distinct cDC subsets. These developmental processes are strongly driven by FLT3L and controlled by several transcription factors (TF; indicated in blue). Monocytes also fully differentiate in the bone marrow but upon entry into lymphoid and non-lymphoid tissues and directed by environmental cues they can acquire features of DCs or MØs. In contrast to DCs and monocytes, most tissue-resident MØs arise from embryonic progenitors and are maintained by self-renewal. Markers commonly used to distinguish pDCs, cDCs, monocyte-derived cells, and MØs in mice are shown. (*) indicates that the specified marker can be expressed on ontogenetically distinct MP subtypes in some instances. (+/−) indicates markers with heterogeneous or tissue-dependent expression on the specified MP sublineage.

The studies discussed above have significantly advanced our understanding of DC ontogeny but have also uncovered some uncertainties (Figure 3). While the bone marrow origin of DCs and monocytes is undisputed, the exact developmental intermediates and branching points between HSCs and DC progenitors remain to be clarified. Current data indicate that lineage imprinting toward DCs and monocytes may occur as early as LMPPs, potentially through epigenetic modification (Figure 3). This realization constitutes a major conceptual shift as it puts in question the existence of a bi-potential MDP and the resulting relatedness of DCs and monocytes. A definitive resolution of this question requires increasingly refined methods to genetically trace single progenitors or select DC and monocyte lineages. Nonetheless, it is clear that cDCs, pDCs, and monocytes can be separated based on their descendance from committed developmental intermediates (Figure 3). Their differentiation is further driven by unique factors indicating that their developmental paths are distinct (Figure 3). In stark contrast to pDCs, cDCs, and monocytes, most tissue MØs arise from embryonic progenitors and are predominantly maintained by self-renewal into adulthood (Figure 3).

Taken together, these data unequivocally establish that DCs, monocytes, and MØs develop as unique cellular entities and although one could argue that most of this knowledge is derived from mouse studies, developmental parallels have been observed in other species (66–73). Despite these advances, we are at a loss for a universal definition of DCs that is readily accessible to experts within and outside the field of MP biology. In light of this recognition, it has been suggested to revise the current nomenclature of MPs into a system that takes cell ontogeny into account when defining subpopulations (6). Such system would greatly aid our understanding of phagocyte biology as it remains uncertain to what extent the cellular origin of DCs, monocytes, and MØs determines the unique functionality of these cells in immunity and/or tissue homeostasis. While global profiling has revealed a role for the local tissue microenvironment in shaping the transcriptional landscape of DCs, monocytes, and MØs from different organs, certain gene signatures and transcriptional features are set by ontogeny (167, 191–193). Therefore, the full functional diversity of DCs, monocytes, and MØs is likely shaped by both nature (ontogeny) and nurture (the environment). Since ontogeny is immutable it provides a more robust common denominator for cell definition that enables deciphering cellular functions without assuming preconceived functional or phenotypic relationships. DC classification based on cell ancestry is a work in progress but its implementation will ultimately yield a more robust and transparent way of cell definition.

## Conflict of Interest Statement

The authors declare that the research was conducted in the absence of any commercial or financial relationships that could be construed as a potential conflict of interest.

## References

[1] Steinman RM Cohn ZA Identification of a novel cell type in peripheral lymphoid organs of mice. I. Morphology, quantitation, tissue distribution. J Exp Med (1973) 137(5):1142–62 10.1084/jem.137.5.1142 4573839 PMC2139237

[2] Steinman RM Witmer MD. Lymphoid dendritic cells are potent stimulators of the primary mixed leukocyte reaction in mice. Proc Natl Acad Sci U S A (1978) 75(10):5132–6. 10.1073/pnas.75.10.5132 154105 PMC336278

[3] Steinman RM Idoyaga J Features of the dendritic cell lineage. Immunol Rev (2010) 234(1):5–17 10.1111/j.0105-2896.2009.00888.x 20193008

[4] van Furth R Cohn ZA. The origin and kinetics of mononuclear phagocytes. J Exp Med (1968) 128(3):415–35. 10.1084/jem.128.3.415 5666958 PMC2138527

[5] van Furth R Cohn ZA Hirsch JG Humphrey JH Spector WG Langevoort HL. The mononuclear phagocyte system: a new classification of macrophages, monocytes, and their precursor cells. Bull World Health Organ (1972) 46(6):845–52. 4538544 PMC2480884

[6] Guilliams M Ginhoux F Jakubzick C Naik SH Onai N Schraml BU Dendritic cells, monocytes and macrophages: a unified nomenclature based on ontogeny. Nat Rev Immunol (2014) 14(8):571–8. 10.1038/nri3712 25033907 PMC4638219

[7] Hashimoto D Miller J Merad M Dendritic cell and macrophage heterogeneity in vivo. Immunity (2011) 35(3):323–35 10.1016/j.immuni.2011.09.007 21943488 PMC4520532

[8] Gordon S Taylor PR Monocyte and macrophage heterogeneity. Nat Rev Immunol (2005) 5(12):953–64 10.1038/nri1733 16322748

[9] van Furth R Identification of mononuclear phagocytes: overview and definitions. In: Adams DO Edelson PJ Koren H, editors. Methods for Studying Mononuclear Phagocytes. New York: Academic Press (1980). p. 243–52.

[10] Schraml BU Reis e Sousa C Defining dendritic cells. Curr Opin Immunol (2015) 32:13–20 10.1016/j.coi.2014.11.001 25553392

[11] Geissmann F Gordon S Hume DA Mowat AM Randolph GJ. Unravelling mononuclear phagocyte heterogeneity. Nat Rev Immunol (2010) 10(6):453–60. 10.1038/nri2784 20467425 PMC3032581

[12] Hume DA. Macrophages as APC and the dendritic cell myth. J Immunol (2008) 181(9):5829–35. 10.4049/jimmunol.181.9.5829 18941170

[13] Hume DA The mononuclear phagocyte system. Curr Opin Immunol (2006) 18(1):49–53 10.1016/j.coi.2005.11.008 16338128

[14] Hume DA Ross IL Himes SR Sasmono RT Wells CA Ravasi T. The mononuclear phagocyte system revisited. J Leukoc Biol (2002) 72(4):621–7. 12377929

[15] Reizis B. Regulation of plasmacytoid dendritic cell development. Curr Opin Immunol (2010) 22(2):206–11. 10.1016/j.coi.2010.01.005 20144853 PMC2854232

[16] Heath WR Carbone FR. Dendritic cell subsets in primary and secondary T cell responses at body surfaces. Nat Immunol (2009) 10(12):1237–44. 10.1038/ni.1822 19915624

[17] Briseño CG Murphy TL Murphy KM. Complementary diversification of dendritic cells and innate lymphoid cells. Curr Opin Immunol (2014) 29C:69–78. 10.1016/j.coi.2014.04.006 24874447 PMC5161034

[18] Merad M Sathe P Helft J Miller J Mortha A. The dendritic cell lineage: ontogeny and function of dendritic cells and their subsets in the steady state and the inflamed setting. Annu Rev Immunol (2013) 31:563–604. 10.1146/annurev-immunol-020711-074950 23516985 PMC3853342

[19] Geissmann F Manz MG Jung S Sieweke MH Merad M Ley K. Development of monocytes, macrophages, and dendritic cells. Science (2010) 327(5966):656–61. 10.1126/science.1178331 20133564 PMC2887389

[20] Nussenzweig MC Steinman RM Witmer MD Gutchinov B. A monoclonal antibody specific for mouse dendritic cells. Proc Natl Acad Sci U S A (1982) 79(1):161–5. 10.1073/pnas.79.1.161 6948298 PMC345682

[21] Steinman RM Kaplan G Witmer MD Cohn ZA. Identification of a novel cell type in peripheral lymphoid organs of mice. V. Purification of spleen dendritic cells, new surface markers, and maintenance in vitro. J Exp Med (1979) 149(1):1–16. 10.1084/jem.149.1.1 762493 PMC2184752

[22] Metlay JP Witmer-Pack MD Agger R Crowley MT Lawless D Steinman RM. The distinct leukocyte integrins of mouse spleen dendritic cells as identified with new hamster monoclonal antibodies. J Exp Med (1990) 171(5):1753–71. 10.1084/jem.171.5.1753 2185332 PMC2187889

[23] Shortman K Naik SH Steady-state and inflammatory dendritic-cell development. Nat Rev Immunol (2007) 7(1):19–30 10.1038/nri1996 17170756

[24] Mildner A Yona S Jung S. A close encounter of the third kind: monocyte-derived cells. Adv Immunol (2013) 120:69–103. 10.1016/B978-0-12-417028-5.00003-X 24070381

[25] Carlens J Wahl B Ballmaier M Bulfone-Paus S Forster R Pabst O. Common gamma-chain-dependent signals confer selective survival of eosinophils in the murine small intestine. J Immunol (2009) 183(9):5600–7. 10.4049/jimmunol.0801581 19843944

[26] Vermaelen K Pauwels R. Accurate and simple discrimination of mouse pulmonary dendritic cell and macrophage populations by flow cytometry: methodology and new insights. Cytometry A (2004) 61(2):170–7. 10.1002/cyto.a.20064 15382026

[27] Probst HC Tschannen K Odermatt B Schwendener R Zinkernagel RM Van Den Broek M. Histological analysis of CD11c-DTR/GFP mice after in vivo depletion of dendritic cells. Clin Exp Immunol (2005) 141(3):398–404. 10.1111/j.1365-2249.2005.02868.x 16045728 PMC1809468

[28] Hochweller K Striegler J Hämmerling GJ Garbi N. A novel CD11c.DTR transgenic mouse for depletion of dendritic cells reveals their requirement for homeostatic proliferation of natural killer cells. Eur J Immunol (2008) 38(10):2776–83. 10.1002/eji.200838659 18825750

[29] Huleatt JW Lefrançois L. Antigen-driven induction of CD11c on intestinal intraepithelial lymphocytes and CD8+ T cells in vivo. J Immunol (1995) 154(11):5684–93. 7751620

[30] Rubtsov AV Rubtsova K Fischer A Meehan RT Gillis JZ Kappler JW Toll-like receptor 7 (TLR7)-driven accumulation of a novel CD11c(+) B-cell population is important for the development of autoimmunity. Blood (2011) 118(5):1305–15. 10.1182/blood-2011-01-331462 21543762 PMC3152497

[31] Drutman SB Kendall JC Trombetta ES. Inflammatory spleen monocytes can upregulate CD11c expression without converting into dendritic cells. J Immunol (2012) 188(8):3603–10. 10.4049/jimmunol.1102741 22442444 PMC4594880

[32] Hebel K Griewank K Inamine A Chang H-D Müller-Hilke B Fillatreau S Plasma cell differentiation in T-independent type 2 immune responses is independent of CD11c(high) dendritic cells. Eur J Immunol (2006) 36(11):2912–9. 10.1002/eji.200636356 17051619

[33] Kim K-W Vallon-Eberhard A Zigmond E Farache J Shezen E Shakhar G In vivo structure/function and expression analysis of the CX3C chemokine fractalkine. Blood (2011) 118(22):e156–67. 10.1182/blood-2011-04-348946 21951685 PMC4507037

[34] Farache J Koren I Milo I Gurevich I Kim KW Zigmond E Luminal bacteria recruit CD103+ dendritic cells into the intestinal epithelium to sample bacterial antigens for presentation. Immunity (2013) 38(3):581–95. 10.1016/j.immuni.2013.01.009 23395676 PMC4115273

[35] Bergstresser PR Sullivan S Streilein JW Tigelaar RE. Origin and function of Thy-1+ dendritic epidermal cells in mice. J Invest Dermatol (1985) 85(1 Suppl):85s–90s. 10.1111/1523-1747.ep12275516 2409184

[36] Schraml BU van Blijswijk J Zelenay S Whitney PG Filby A Acton SE Genetic tracing via DNGR-1 expression history defines dendritic cells as a hematopoietic lineage. Cell (2013) 154(4):843–58. 10.1016/j.cell.2013.07.014 23953115

[37] Satpathy AT Kc W Albring JC Edelson BT Kretzer NM Bhattacharya D Zbtb46 expression distinguishes classical dendritic cells and their committed progenitors from other immune lineages. J Exp Med (2012) 209(6):1135–52. 10.1084/jem.20120030 22615127 PMC3371733

[38] Meredith MM Liu K Darrasse-Jèze G Kamphorst AO Schreiber HA Guermonprez P Expression of the zinc finger transcription factor zDC (Zbtb46, Btbd4) defines the classical dendritic cell lineage. J Exp Med (2012) 209(6):1153–65. 10.1084/jem.20112675 22615130 PMC3371731

[39] Mashayekhi M Sandau MM Dunay IR Frickel EM Khan A Goldszmid RS CD8α(+) dendritic cells are the critical source of interleukin-12 that controls acute infection by *Toxoplasma gondii* tachyzoites. Immunity (2011) 35(2):249–59. 10.1016/j.immuni.2011.08.008 21867928 PMC3171793

[40] Reis e Sousa C Hieny S Scharton-Kersten T Jankovic D Charest H Germain RN In vivo microbial stimulation induces rapid CD40 ligand-independent production of interleukin 12 by dendritic cells and their redistribution to T cell areas. J Exp Med (1997) 186(11):1819–29. 10.1084/jem.186.11.1819 9382881 PMC2199158

[41] Whitney PG Bar E Osorio F Rogers NC Schraml BU Deddouche S Syk signaling in dendritic cells orchestrates innate resistance to systemic fungal infection. PLoS Pathog (2014) 10(7):e1004276. 10.1371/journal.ppat.1004276 25033445 PMC4102599

[42] Satpathy AT Briseño CG Lee JS Ng D Manieri NA Kc W Notch2-dependent classical dendritic cells orchestrate intestinal immunity to attaching-and-effacing bacterial pathogens. Nat Immunol (2013) 14(9):937–48. 10.1038/ni.2679 23913046 PMC3788683

[43] Kinnebrew MA Buffie CG Diehl GE Zenewicz LA Leiner I Hohl TM Interleukin 23 production by intestinal CD103(+)CD11b(+) dendritic cells in response to bacterial flagellin enhances mucosal innate immune defense. Immunity (2012) 36(2):276–87. 10.1016/j.immuni.2011.12.011 22306017 PMC3288454

[44] Arora P Baena A Yu KOA Saini NK Kharkwal SS Goldberg MF A single subset of dendritic cells controls the cytokine bias of natural killer T cell responses to diverse glycolipid antigens. Immunity (2014) 40(1):105–16. 10.1016/j.immuni.2013.12.004 24412610 PMC3895174

[45] Jakubzick C Gautier EL Gibbings SL Sojka DK Schlitzer A Johnson TE Minimal differentiation of classical monocytes as they survey steady-state tissues and transport antigen to lymph nodes. Immunity (2013) 39(3):599–610. 10.1016/j.immuni.2013.08.007 24012416 PMC3820017

[46] Plantinga M Guilliams M Vanheerswynghels M Deswarte K Branco-Madeira F Toussaint W Conventional and monocyte-derived CD11b(+) dendritic cells initiate and maintain T helper 2 cell-mediated immunity to house dust mite allergen. Immunity (2013) 38(2):322–35. 10.1016/j.immuni.2012.10.016 23352232

[47] Pozzi LA Maciaszek JW Rock KL. Both dendritic cells and macrophages can stimulate naive CD8 T cells in vivo to proliferate, develop effector function, and differentiate into memory cells. J Immunol (2005) 175(4):2071–81. 10.4049/jimmunol.175.4.2071 16081773

[48] McGovern N Schlitzer A Gunawan M Jardine L Shin A Poyner E Human dermal CD14+ cells are a transient population of monocyte-derived macrophages. Immunity (2014) 41(3):465–77. 10.1016/j.immuni.2014.08.006 25200712 PMC4175180

[49] Naik SH Perié L Swart E Gerlach C van Rooij N de Boer RJ Diverse and heritable lineage imprinting of early haematopoietic progenitors. Nature (2013) 496(7444):229–32. 10.1038/nature12013 23552896

[50] Yona S Kim K-W Wolf Y Mildner A Varol D Breker M Fate mapping reveals origins and dynamics of monocytes and tissue macrophages under homeostasis. Immunity (2013) 38(1):79–91. 10.1016/j.immuni.2012.12.001 23273845 PMC3908543

[51] Schulz C Gomez Perdiguero E Chorro L Szabo-Rogers H Cagnard N Kierdorf K A lineage of myeloid cells independent of Myb and hematopoietic stem cells. Science (2012) 336(6077):86–90. 10.1126/science.1219179 22442384

[52] Hettinger J Richards DM Hansson J Barra MM Joschko A-C Krijgsveld J Origin of monocytes and macrophages in a committed progenitor. Nat Immunol (2013) 14(8):821–30. 10.1038/ni.2638 23812096

[53] Haniffa M Ginhoux F Wang X-N Bigley V Abel M Dimmick I Differential rates of replacement of human dermal dendritic cells and macrophages during hematopoietic stem cell transplantation. J Exp Med (2009) 206(2):371–85. 10.1084/jem.20081633 19171766 PMC2646566

[54] Hashimoto D Chow A Noizat C Teo P Beasley MB Leboeuf M Tissue-resident macrophages self-maintain locally throughout adult life with minimal contribution from circulating monocytes. Immunity (2013) 38(4):792–804. 10.1016/j.immuni.2013.04.004 23601688 PMC3853406

[55] Iwasaki H Akashi K Myeloid lineage commitment from the hematopoietic stem cell. Immunity (2007) 26(6):726–40 10.1016/j.immuni.2007.06.004 17582345

[56] Fogg DK Sibon C Miled C Jung S Aucouturier P Littman DR A clonogenic bone marrow progenitor specific for macrophages and dendritic cells. Science (2006) 311(5757):83–7. 10.1126/science.1117729 16322423

[57] Naik SH Sathe P Park H-Y Metcalf D Proietto AI Dakic A Development of plasmacytoid and conventional dendritic cell subtypes from single precursor cells derived in vitro and in vivo. Nat Immunol (2007) 8(11):1217–26. 10.1038/ni1522 17922015

[58] Onai N Obata-Onai A Schmid MA Ohteki T Jarrossay D Manz MG. Identification of clonogenic common Flt3+M-CSFR+ plasmacytoid and conventional dendritic cell progenitors in mouse bone marrow. Nat Immunol (2007) 8(11):1207–16. 10.1038/ni1518 17922016

[59] Shortman K Sathe P Vremec D Naik S O’Keeffe M. Plasmacytoid dendritic cell development. Adv Immunol (2013) 120:105–26. 10.1016/B978-0-12-417028-5.00004-1 24070382

[60] Naik SH Metcalf D Van Nieuwenhuijze A Wicks I Wu L O’Keeffe M Intrasplenic steady-state dendritic cell precursors that are distinct from monocytes. Nat Immunol (2006) 7(6):663–71. 10.1038/ni1340 16680143

[61] Liu K Victora GD Schwickert TA Guermonprez P Meredith MM Yao K In vivo analysis of dendritic cell development and homeostasis. Science (2009) 324(5925):392–7. 10.1126/science.1170540 19286519 PMC2803315

[62] Liu K Waskow C Liu X Yao K Hoh J Nussenzweig M. Origin of dendritic cells in peripheral lymphoid organs of mice. Nat Immunol (2007) 8(6):578–83. 10.1038/ni1462 17450143

[63] Ginhoux F Liu K Helft J Bogunovic M Greter M Hashimoto D The origin and development of nonlymphoid tissue CD103+ DCs. J Exp Med (2009) 206(13):3115–30. 10.1084/jem.20091756 20008528 PMC2806447

[64] Serbina NV Salazar-Mather TP Biron CA Kuziel WA Pamer EG. TNF/iNOS-producing dendritic cells mediate innate immune defense against bacterial infection. Immunity (2003) 19(1):59–70. 10.1016/S1074-7613(03)00171-7 12871639

[65] Haniffa M Collin M Ginhoux F. Ontogeny and functional specialization of dendritic cells in human and mouse. Adv Immunol (2013) 120:1–49. 10.1016/B978-0-12-417028-5.00001-6 24070379

[66] Guzylack-Piriou L Alves MP McCullough KC Summerfield A. Porcine Flt3 ligand and its receptor: generation of dendritic cells and identification of a new marker for porcine dendritic cells. Dev Comp Immunol (2010) 34(4):455–64. 10.1016/j.dci.2009.12.006 20015454

[67] Cisse B Caton ML Lehner M Maeda T Scheu S Locksley R Transcription factor E2-2 is an essential and specific regulator of plasmacytoid dendritic cell development. Cell (2008) 135(1):37–48. 10.1016/j.cell.2008.09.016 18854153 PMC2631034

[68] Maraskovsky E Daro E Roux E Teepe M Maliszewski CR Hoek J In vivo generation of human dendritic cell subsets by Flt3 ligand. Blood (2000) 96(3):878–84. 10910900

[69] Hambleton S Salem S Bustamante J Bigley V Boisson-Dupuis S Azevedo J IRF8 mutations and human dendritic-cell immunodeficiency. N Engl J Med (2011) 365(2):127–38. 10.1056/NEJMoa1100066 21524210 PMC3136554

[70] Harada S Kimura T Fujiki H Nakagawa H Ueda Y Itoh T Flt3 ligand promotes myeloid dendritic cell differentiation of human hematopoietic progenitor cells: possible application for cancer immunotherapy. Int J Oncol (2007) 30(6):1461–8. 10.3892/ijo.30.6.1461 17487367

[71] Poulin LF Reyal Y Uronen-Hansson H Schraml BU Sancho D Murphy KM DNGR-1 is a specific and universal marker of mouse and human Batf3-dependent dendritic cells in lymphoid and nonlymphoid tissues. Blood (2012) 119(25):6052–62. 10.1182/blood-2012-01-406967 22442345

[72] Breton G Lee J Zhou YJ Schreiber JJ Keler T Puhr S Circulating precursors of human CD1c+ and CD141+ dendritic cells. J Exp Med (2015) 212(3):401–13. 10.1084/jem.20141441 25687281 PMC4354370

[73] Lee J Breton G Oliveira TYK Zhou YJ Aljoufi A Puhr S Restricted dendritic cell and monocyte progenitors in human cord blood and bone marrow. J Exp Med (2015) 212(3):385–99. 10.1084/jem.20141442 25687283 PMC4354373

[74] Traver D Akashi K Manz M Merad M Miyamoto T Engleman EG Development of CD8alpha-positive dendritic cells from a common myeloid progenitor. Science (2000) 290(5499):2152–4. 10.1126/science.290.5499.2152 11118150

[75] Schlenner SM Madan V Busch K Tietz A Läufle C Costa C Fate mapping reveals separate origins of T cells and myeloid lineages in the thymus. Immunity (2010) 32(3):426–36. 10.1016/j.immuni.2010.03.005 20303297

[76] Luche H Ardouin L Teo P See P Henri S Merad M The earliest intrathymic precursors of CD8α(+) thymic dendritic cells correspond to myeloid-type double-negative 1c cells. Eur J Immunol (2011) 41(8):2165–75. 10.1002/eji.201141728 21630253 PMC4291128

[77] Sathe P Vremec D Wu L Corcoran L Shortman K. Convergent differentiation: myeloid and lymphoid pathways to murine plasmacytoid dendritic cells. Blood (2013) 121(1):11–9. 10.1182/blood-2012-02-413336 23053574

[78] Sathe P Metcalf D Vremec D Naik SH Langdon WY Huntington ND Lymphoid tissue and plasmacytoid dendritic cells and macrophages do not share a common macrophage-dendritic cell-restricted progenitor. Immunity (2014) 41(1):104–15. 10.1016/j.immuni.2014.05.020 25035955

[79] Onai N Kurabayashi K Hosoi-Amaike M Toyama-Sorimachi N Matsushima K Inaba K A clonogenic progenitor with prominent plasmacytoid dendritic cell developmental potential. Immunity (2013) 38(5):943–57. 10.1016/j.immuni.2013.04.006 23623382

[80] Kretzschmar K Watt FM. Lineage tracing. Cell (2012) 148(1–2):33–45. 10.1016/j.cell.2012.01.002 22265400

[81] Naik SH Schumacher TN Perié L. Cellular barcoding: a technical appraisal. Exp Hematol (2014) 42(8):598–608. 10.1016/j.exphem.2014.05.003 24996012

[82] Bar-On L Jung S. Defining dendritic cells by conditional and constitutive cell ablation. Immunol Rev (2010) 234(1):76–89. 10.1111/j.0105-2896.2009.00875.x 20193013

[83] Plett PA Frankovitz SM Orschell-Traycoff CM. In vivo trafficking, cell cycle activity, and engraftment potential of phenotypically defined primitive hematopoietic cells after transplantation into irradiated or nonirradiated recipients. Blood (2002) 100(10):3545–52. 10.1182/blood.V100.10.3545 12411318

[84] Varol C Landsman L Fogg DK Greenshtein L Gildor B Margalit R Monocytes give rise to mucosal, but not splenic, conventional dendritic cells. J Exp Med (2007) 204(1):171–80. 10.1084/jem.20061011 17190836 PMC2118434

[85] Waskow C Liu K Darrasse-Jèze G Guermonprez P Ginhoux F Merad M The receptor tyrosine kinase Flt3 is required for dendritic cell development in peripheral lymphoid tissues. Nat Immunol (2008) 9(6):676–83. 10.1038/ni.1615 18469816 PMC2746085

[86] Auffray C Fogg DK Narni-Mancinelli E Senechal B Trouillet C Saederup N CX3CR1+ CD115+ CD135+ common macrophage/DC precursors and the role of CX3CR1 in their response to inflammation. J Exp Med (2009) 206(3):595–606. 10.1084/jem.20081385 19273628 PMC2699130

[87] Schlitzer A Loschko J Mair K Vogelmann R Henkel L Einwächter H Identification of CCR9- murine plasmacytoid DC precursors with plasticity to differentiate into conventional DCs. Blood (2011) 117(24):6562–70. 10.1182/blood-2010-12-326678 21508410

[88] Sallusto F Lanzavecchia A. Efficient presentation of soluble antigen by cultured human dendritic cells is maintained by granulocyte/macrophage colony-stimulating factor plus interleukin 4 and downregulated by tumor necrosis factor alpha. J Exp Med (1994) 179(4):1109–18. 10.1084/jem.179.4.1109 8145033 PMC2191432

[89] Romani N Gruner S Brang D Kämpgen E Lenz A Trockenbacher B Proliferating dendritic cell progenitors in human blood. J Exp Med (1994) 180(1):83–93 10.1084/jem.180.1.83 8006603 PMC2191538

[90] Greter M Helft J Chow A Hashimoto D Mortha A Agudo-Cantero J GM-CSF controls nonlymphoid tissue dendritic cell homeostasis but is dispensable for the differentiation of inflammatory dendritic cells. Immunity (2012) 36(6):1031–46. 10.1016/j.immuni.2012.03.027 22749353 PMC3498051

[91] Bogunovic M Ginhoux F Helft J Shang L Hashimoto D Greter M Origin of the lamina propria dendritic cell network. Immunity (2009) 31(3):513–25. 10.1016/j.immuni.2009.08.010 19733489 PMC2778256

[92] Varol C Vallon-Eberhard A Elinav E Aychek T Shapira Y Luche H Intestinal lamina propria dendritic cell subsets have different origin and functions. Immunity (2009) 31(3):502–12. 10.1016/j.immuni.2009.06.025 19733097

[93] Tamoutounour S Henri S Lelouard H de Bovis B de Haar C van der Woude CJ CD64 distinguishes macrophages from dendritic cells in the gut and reveals the Th1-inducing role of mesenteric lymph node macrophages during colitis. Eur J Immunol (2012) 42(12):3150–66. 10.1002/eji.201242847 22936024

[94] Langlet C Tamoutounour S Henri S Luche H Ardouin L Grégoire C CD64 expression distinguishes monocyte-derived and conventional dendritic cells and reveals their distinct role during intramuscular immunization. J Immunol (2012) 188(4):1751–60. 10.4049/jimmunol.1102744 22262658

[95] Scott CL Bain CC Wright PB Sichien D Kotarsky K Persson EK CCR2(+)CD103(-) intestinal dendritic cells develop from DC-committed precursors and induce interleukin-17 production by T cells. Mucosal Immunol (2014) 8(2):327–39. 10.1038/mi.2014.70 25138666 PMC4270738

[96] Merad M Manz MG Karsunky H Wagers A Peters W Charo I Langerhans cells renew in the skin throughout life under steady-state conditions. Nat Immunol (2002) 3(12):1135–41. 10.1038/ni852 12415265 PMC4727838

[97] Ginhoux F Tacke F Angeli V Bogunovic M Loubeau M Dai X-M Langerhans cells arise from monocytes in vivo. Nat Immunol (2006) 7(3):265–73. 10.1038/ni1307 16444257 PMC4727824

[98] Merad M Ginhoux F Collin M. Origin, homeostasis and function of Langerhans cells and other langerin-expressing dendritic cells. Nat Rev Immunol (2008) 8(12):935–47. 10.1038/nri2455 19029989

[99] Romani N Clausen BE Stoitzner P. Langerhans cells and more: langerin-expressing dendritic cell subsets in the skin. Immunol Rev (2010) 234(1):120–41. 10.1111/j.0105-2896.2009.00886.x 20193016 PMC2907488

[100] Ginhoux F Merad M. Ontogeny and homeostasis of Langerhans cells. Immunol Cell Biol (2010) 88(4):387–92. 10.1038/icb.2010.38 20309014

[101] Hoeffel G Wang Y Greter M See P Teo P Malleret B Adult Langerhans cells derive predominantly from embryonic fetal liver monocytes with a minor contribution of yolk sac-derived macrophages. J Exp Med (2012) 209(6):1167–81. 10.1084/jem.20120340 22565823 PMC3371735

[102] Chorro L Sarde A Li M Woollard KJ Chambon P Malissen B Langerhans cell (LC) proliferation mediates neonatal development, homeostasis, and inflammation-associated expansion of the epidermal LC network. J Exp Med (2009) 206(13):3089–100. 10.1084/jem.20091586 19995948 PMC2806478

[103] Hume DA Applications of myeloid-specific promoters in transgenic mice support in vivo imaging and functional genomics but do not support the concept of distinct macrophage and dendritic cell lineages or roles in immunity. J Leukoc Biol (2011) 89(4):525–38 10.1189/jlb.0810472 21169519

[104] Lindquist RL Shakhar G Dudziak D Wardemann H Eisenreich T Dustin ML Visualizing dendritic cell networks in vivo. Nat Immunol (2004) 5(12):1243–50 10.1038/ni1139 15543150

[105] Thornton EE Looney MR Bose O Sen D Sheppard D Locksley R Spatiotemporally separated antigen uptake by alveolar dendritic cells and airway presentation to T cells in the lung. J Exp Med (2012) 209(6):1183–99. 10.1084/jem.20112667 22585735 PMC3371730

[106] Choi J-H Do Y Cheong C Koh H Boscardin SB Oh Y-S Identification of antigen-presenting dendritic cells in mouse aorta and cardiac valves. J Exp Med (2009) 206(3):497–505. 10.1084/jem.20082129 19221394 PMC2699134

[107] Khanna KM Blair DA Vella AT McSorley SJ Datta SK Lefrançois L. T cell and APC dynamics in situ control the outcome of vaccination. J Immunol (2010) 185(1):239–52. 10.4049/jimmunol.0901047 20530268 PMC2997353

[108] Bennett CL Clausen BE. DC ablation in mice: promises, pitfalls, and challenges. Trends Immunol (2007) 28(12):525–31. 10.1016/j.it.2007.08.011 17964853

[109] Jung S Unutmaz D Wong P Sano G-I de los Santos K Sparwasser T In vivo depletion of CD11c+ dendritic cells abrogates priming of CD8+ T cells by exogenous cell-associated antigens. Immunity (2002) 17(2):211–20. 10.1016/S1074-7613(02)00365-5 12196292 PMC3689299

[110] Tittel AP Heuser C Ohliger C Knolle PA Engel DR Kurts C. Kidney dendritic cells induce innate immunity against bacterial pyelonephritis. J Am Soc Nephrol (2011) 22(8):1435–41. 10.1681/ASN.2010101072 21757770 PMC3148698

[111] Bar-On L Birnberg T Kim K-W Jung S. Dendritic cell-restricted CD80/86 deficiency results in peripheral regulatory T-cell reduction but is not associated with lymphocyte hyperactivation. Eur J Immunol (2011) 41(2):291–8. 10.1002/eji.201041169 21267999

[112] van Blijswijk J Schraml BU Reis e Sousa C. Advantages and limitations of mouse models to deplete dendritic cells. Eur J Immunol (2013) 43(1):22–6. 10.1002/eji.201243022 23322690

[113] Racine R Chatterjee M Winslow GM. CD11c expression identifies a population of extrafollicular antigen-specific splenic plasmablasts responsible for CD4 T-independent antibody responses during intracellular bacterial infection. J Immunol (2008) 181(2):1375–85. 10.4049/jimmunol.181.2.1375 18606692 PMC2645789

[114] Brocker T Riedinger M Karjalainen K Driving gene expression specifically in dendritic cells. Adv Exp Med Biol (1997) 417:55–7 10.1007/978-1-4757-9966-8_9 9286337

[115] Tittel AP Heuser C Ohliger C Llanto C Yona S Hämmerling GJ Functionally relevant neutrophilia in CD11c diphtheria toxin receptor transgenic mice. Nat Methods (2012) 9(4):385–90. 10.1038/nmeth.1905 22367054

[116] Bar-On L Jung S. Defining in vivo dendritic cell functions using CD11c-DTR transgenic mice. Methods Mol Biol (2010) 595:429–42. 10.1007/978-1-60761-421-0_28 19941129

[117] Jiao J Dragomir A-C Kocabayoglu P Rahman AH Chow A Hashimoto D Central role of conventional dendritic cells in regulation of bone marrow release and survival of neutrophils. J Immunol (2014) 192(7):3374–82. 10.4049/jimmunol.1300237 24591364 PMC4144807

[118] Gardner JM Metzger TC McMahon EJ Au-Yeung BB Krawisz AK Lu W Extrathymic aire-expressing cells are a distinct bone marrow-derived population that induce functional inactivation of CD4+ T Cells. Immunity (2013) 39(3):560–72. 10.1016/j.immuni.2013.08.005 23993652 PMC3804105

[119] Epelman S Lavine KJ Beaudin AE Sojka DK Carrero JA Calderon B Embryonic and adult-derived resident cardiac macrophages are maintained through distinct mechanisms at steady state and during inflammation. Immunity (2014) 40(1):91–104. 10.1016/j.immuni.2013.11.019 24439267 PMC3923301

[120] Broz ML Binnewies M Boldajipour B Nelson AE Pollack JL Erle DJ Dissecting the tumor myeloid compartment reveals rare activating antigen-presenting cells critical for T cell immunity. Cancer Cell (2014) 26(5):638–52. 10.1016/j.ccell.2014.09.007 25446897 PMC4254577

[121] Ferris ST Carrero JA Mohan JF Calderon B Murphy KM Unanue ER. A minor subset of Batf3-dependent antigen-presenting cells in islets of langerhans is essential for the development of autoimmune diabetes. Immunity (2014) 41(4):657–69. 10.1016/j.immuni.2014.09.012 25367577 PMC4220295

[122] Segura E Touzot M Bohineust A Cappuccio A Chiocchia G Hosmalin A Human inflammatory dendritic cells induce Th17 cell differentiation. Immunity (2013) 38(2):336–48. 10.1016/j.immuni.2012.10.018 23352235

[123] Meredith MM Liu K Kamphorst AO Idoyaga J Yamane A Guermonprez P Zinc finger transcription factor zDC is a negative regulator required to prevent activation of classical dendritic cells in the steady state. J Exp Med (2012) 209(9):1583–93. 10.1084/jem.20121003 22851594 PMC3428942

[124] Hildner K Edelson BT Purtha WE Diamond M Matsushita H Kohyama M Batf3 deficiency reveals a critical role for CD8alpha+ dendritic cells in cytotoxic T cell immunity. Science (2008) 322(5904):1097–100. 10.1126/science.1164206 19008445 PMC2756611

[125] Edelson BT Kc W Juang R Kohyama M Benoit LA Klekotka PA Peripheral CD103+ dendritic cells form a unified subset developmentally related to CD8alpha+ conventional dendritic cells. J Exp Med (2010) 207(4):823–36. 10.1084/jem.20091627 20351058 PMC2856032

[126] Schiavoni G Mattei F Sestili P Borghi P Venditti M Morse HC ICSBP is essential for the development of mouse type I interferon-producing cells and for the generation and activation of CD8alpha(+) dendritic cells. J Exp Med (2002) 196(11):1415–25. 10.1084/jem.20021263 12461077 PMC2194263

[127] Hacker C Kirsch RD Ju X-S Hieronymus T Gust TC Kuhl C Transcriptional profiling identifies Id2 function in dendritic cell development. Nat Immunol (2003) 4(4):380–6. 10.1038/ni903 12598895

[128] Kashiwada M Pham NL Pewe LL Harty JT Rothman PB. NFIL3/E4BP4 is a key transcription factor for CD8alpha(+) dendritic cell development. Blood (2011) 117(23):6193–7. 10.1182/blood-2010-07-295873 21474667 PMC3122942

[129] Suzuki S Honma K Matsuyama T Suzuki K Toriyama K Akitoyo I Critical roles of interferon regulatory factor 4 in CD11bhighCD8alpha- dendritic cell development. Proc Natl Acad Sci U S A (2004) 101(24):8981–6. 10.1073/pnas.0402139101 15184678 PMC428458

[130] Caton ML Smith-Raska MR Reizis B. Notch-RBP-J signaling controls the homeostasis of CD8- dendritic cells in the spleen. J Exp Med (2007) 204(7):1653–64. 10.1084/jem.20062648 17591855 PMC2118632

[131] Lewis KL Caton ML Bogunovic M Greter M Grajkowska LT Ng D Notch2 receptor signaling controls functional differentiation of dendritic cells in the spleen and intestine. Immunity (2011) 35(5):780–91. 10.1016/j.immuni.2011.08.013 22018469 PMC3225703

[132] Schlitzer A McGovern N Teo P Zelante T Atarashi K Low D IRF4 transcription factor-dependent CD11b+ dendritic cells in human and mouse control mucosal IL-17 cytokine responses. Immunity (2013) 38(5):970–83. 10.1016/j.immuni.2013.04.011 23706669 PMC3666057

[133] Persson EK Uronen-Hansson H Semmrich M Rivollier A Hägerbrand K Marsal J IRF4 transcription-factor-dependent CD103(+)CD11b(+) dendritic cells drive mucosal T helper 17 cell differentiation. Immunity (2013) 38(5):958–69. 10.1016/j.immuni.2013.03.009 23664832

[134] Tamura T Tailor P Yamaoka K Kong HJ Tsujimura H O’shea JJ IFN regulatory factor-4 and -8 govern dendritic cell subset development and their functional diversity. J Immunol (2005) 174(5):2573–81. 10.4049/jimmunol.174.5.2573 15728463

[135] Wu L D’Amico A Winkel KD Suter M Lo D Shortman K. RelB is essential for the development of myeloid-related CD8alpha- dendritic cells but not of lymphoid-related CD8alpha+ dendritic cells. Immunity (1998) 9(6):839–47. 10.1016/S1074-7613(00)80649-4 9881974

[136] Guerriero A Langmuir PB Spain LM Scott EW. PU.1 is required for myeloid-derived but not lymphoid-derived dendritic cells. Blood (2000) 95(3):879–85. 10648399

[137] McKenna HJ Stocking KL Miller RE Brasel K De Smedt T Maraskovsky E Mice lacking flt3 ligand have deficient hematopoiesis affecting hematopoietic progenitor cells, dendritic cells, and natural killer cells. Blood (2000) 95(11):3489–97. 10828034

[138] Kohyama M Ise W Edelson BT Wilker PR Hildner K Mejia C Role for Spi-C in the development of red pulp macrophages and splenic iron homeostasis. Nature (2009) 457(7227):318–21. 10.1038/nature07472 19037245 PMC2756102

[139] Wang Y Szretter KJ Vermi W Gilfillan S Rossini C Cella M IL-34 is a tissue-restricted ligand of CSF1R required for the development of Langerhans cells and microglia. Nat Immunol (2012) 13(8):753–60. 10.1038/ni.2360 22729249 PMC3941469

[140] Greter M Lelios I Pelczar P Hoeffel G Price J Leboeuf M Stroma-derived interleukin-34 controls the development and maintenance of langerhans cells and the maintenance of microglia. Immunity (2012) 37(6):1050–60. 10.1016/j.immuni.2012.11.001 23177320 PMC4291117

[141] Wiktor-Jedrzejczak WW Ahmed A Szczylik C Skelly RR. Hematological characterization of congenital osteopetrosis in op/op mouse. Possible mechanism for abnormal macrophage differentiation. J Exp Med (1982) 156(5):1516–27. 10.1084/jem.156.5.1516 7130905 PMC2186832

[142] Ghosh HS Cisse B Bunin A Lewis KL Reizis B. Continuous expression of the transcription factor e2-2 maintains the cell fate of mature plasmacytoid dendritic cells. Immunity (2010) 33(6):905–16. 10.1016/j.immuni.2010.11.023 21145760 PMC3010277

[143] Helft J Manicassamy B Guermonprez P Hashimoto D Silvin A Agudo J Cross-presenting CD103+ dendritic cells are protected from influenza virus infection. J Clin Invest (2012) 122(11):4037–47. 10.1172/JCI60659 23041628 PMC3484433

[144] Desch AN Randolph GJ Murphy K Gautier EL Kedl RM Lahoud MH CD103+ pulmonary dendritic cells preferentially acquire and present apoptotic cell-associated antigen. J Exp Med (2011) 208(9):1789–97. 10.1084/jem.20110538 21859845 PMC3171085

[145] Bedoui S Whitney PG Waithman J Eidsmo L Wakim L Caminschi I Cross-presentation of viral and self antigens by skin-derived CD103+ dendritic cells. Nat Immunol (2009) 10(5):488–95. 10.1038/ni.1724 19349986

[146] Murphy KM. Transcriptional control of dendritic cell development. Adv Immunol (2013) 120:239–67. 10.1016/B978-0-12-417028-5.00009-0 24070387

[147] Edelson BT Bradstreet TR Kc W Hildner K Herzog JW Sim J Batf3-dependent CD11b(low/-) peripheral dendritic cells are GM-CSF-independent and are not required for Th cell priming after subcutaneous immunization. PLoS One (2011) 6(10):e25660. 10.1371/journal.pone.0025660 22065991 PMC3196467

[148] Tussiwand R Lee W-L Murphy TL Mashayekhi M Wumesh KC Albring JC Compensatory dendritic cell development mediated by BATF-IRF interactions. Nature (2012) 490(7421):502–7. 10.1038/nature11531 22992524 PMC3482832

[149] Seillet C Jackson JT Markey KA Brady HJM Hill GR MacDonald KPA CD8α+ DCs can be induced in the absence of transcription factors Id2, Nfil3, and Batf3. Blood (2013) 121(9):1574–83. 10.1182/blood-2012-07-445650 23297132

[150] Contreras V Urien C Guiton R Alexandre Y Vu Manh T-P Andrieu T Existence of CD8α-like dendritic cells with a conserved functional specialization and a common molecular signature in distant mammalian species. J Immunol (2010) 185(6):3313–25. 10.4049/jimmunol.1000824 20702727

[151] Marquet F Bonneau M Pascale F Urien C Kang C Schwartz-Cornil I Characterization of dendritic cells subpopulations in skin and afferent lymph in the swine model. PLoS One (2011) 6(1):e16320. 10.1371/journal.pone.0016320 21298011 PMC3029332

[152] Vu Manh TP Marty H Sibille P Le Vern Y Kaspers B Dalod M Existence of conventional dendritic cells in Gallus gallus revealed by comparative gene expression profiling. J Immunol (2014) 192(10):4510–7. 10.4049/jimmunol.1303405 24740508

[153] Hubert FX Voisine C Louvet C Heslan M Josien R Rat plasmacytoid dendritic cells are an abundant subset of MHC class II+ CD4+CD11b-OX62- and type I IFN-producing cells that exhibit selective expression of toll-like receptors 7 and 9 and strong responsiveness to CpG. J Immunol (2004) 172(12):7485–94 10.4049/jimmunol.172.12.7485 15187127

[154] Sathaliyawala T O’Gorman WE Greter M Bogunovic M Konjufca V Hou ZE Mammalian target of rapamycin controls dendritic cell development downstream of Flt3 ligand signaling. Immunity (2010) 33(4):597–606. 10.1016/j.immuni.2010.09.012 20933441 PMC2966531

[155] Brasel K McKenna HJ Morrissey PJ Charrier K Morris AE Lee CC Hematologic effects of flt3 ligand in vivo in mice. Blood (1996) 88(6):2004–12. 8822919

[156] Maraskovsky E Brasel K Teepe M Roux ER Lyman SD Shortman K Dramatic increase in the numbers of functionally mature dendritic cells in Flt3 ligand-treated mice: multiple dendritic cell subpopulations identified. J Exp Med (1996) 184(5):1953–62. 10.1084/jem.184.5.1953 8920882 PMC2192888

[157] Pulendran B Banchereau J Burkeholder S Kraus E Guinet E Chalouni C Flt3-ligand and granulocyte colony-stimulating factor mobilize distinct human dendritic cell subsets in vivo. J Immunol (2000) 165(1):566–72. 10.4049/jimmunol.165.1.566 10861097

[158] Naik SH Proietto AI Wilson NS Dakic A Schnorrer P Fuchsberger M Cutting edge: generation of splenic CD8+ and CD8- dendritic cell equivalents in Fms-like tyrosine kinase 3 ligand bone marrow cultures. J Immunol (2005) 174(11):6592–7. 10.4049/jimmunol.174.11.6592 15905497

[159] Poulin LF Salio M Griessinger E Anjos-Afonso F Craciun L Chen J-L Characterization of human DNGR-1+ BDCA3+ leukocytes as putative equivalents of mouse CD8alpha+ dendritic cells. J Exp Med (2010) 207(6):1261–71. 10.1084/jem.20092618 20479117 PMC2882845

[160] Tussiwand R Onai N Mazzucchelli L Manz MG. Inhibition of natural type I IFN-producing and dendritic cell development by a small molecule receptor tyrosine kinase inhibitor with Flt3 affinity. J Immunol (2005) 175(6):3674–80. 10.4049/jimmunol.175.6.3674 16148112

[161] Whartenby KA Calabresi PA McCadden E Nguyen B Kardian D Wang T Inhibition of FLT3 signaling targets DCs to ameliorate autoimmune disease. Proc Natl Acad Sci U S A (2005) 102(46):16741–6. 10.1073/pnas.0506088102 16272221 PMC1283812

[162] Jenkins SJ Hume DA Homeostasis in the mononuclear phagocyte system. Trends Immunol (2014) 35(8):358–67 10.1016/j.it.2014.06.006 25047416

[163] Mackarehtschian K Hardin JD Moore KA Boast S Goff SP Lemischka IR. Targeted disruption of the flk2/flt3 gene leads to deficiencies in primitive hematopoietic progenitors. Immunity (1995) 3(1):147–61. 10.1016/1074-7613(95)90167-1 7621074

[164] Kingston D Schmid MA Onai N Obata-Onai A Baumjohann D Manz MG. The concerted action of GM-CSF and Flt3-ligand on in vivo dendritic cell homeostasis. Blood (2009) 114(4):835–43. 10.1182/blood-2009-02-206318 19465690

[165] Kim S-W Choi S-M Choo YS Kim I-K Song B-W Kim H-S. Flt3 ligand induces monocyte proliferation and enhances the function of monocyte-derived dendritic cells in vitro. J Cell Physiol (2014). 10.1002/jcp.24824 25215878

[166] Xu Y Zhan Y Lew AM Naik SH Kershaw MH. Differential development of murine dendritic cells by GM-CSF versus Flt3 ligand has implications for inflammation and trafficking. J Immunol (2007) 179(11):7577–84. 10.4049/jimmunol.179.11.7577 18025203

[167] Miller JC Brown BD Shay T Gautier EL Jojic V Cohain A Deciphering the transcriptional network of the dendritic cell lineage. Nat Immunol (2012) 13(9):888–99. 10.1038/ni.2370 22797772 PMC3985403

[168] MacDonald KPA Rowe V Bofinger HM Thomas R Sasmono T Hume DA The colony-stimulating factor 1 receptor is expressed on dendritic cells during differentiation and regulates their expansion. J Immunol (2005) 175(3):1399–405. 10.4049/jimmunol.175.3.1399 16034075

[169] Dai XM Ryan GR Hapel AJ Dominguez MG Russell RG Kapp S Targeted disruption of the mouse colony-stimulating factor 1 receptor gene results in osteopetrosis, mononuclear phagocyte deficiency, increased primitive progenitor cell frequencies, and reproductive defects. Blood (2002) 99(1):111–20. 10.1182/blood.V99.1.111 11756160

[170] Fancke B Suter M Hochrein H O’Keeffe M. M-CSF: a novel plasmacytoid and conventional dendritic cell poietin. Blood (2008) 111(1):150–9. 10.1182/blood-2007-05-089292 17916748

[171] Tagliani E Shi C Nancy P Tay C-S Pamer EG Erlebacher A. Coordinate regulation of tissue macrophage and dendritic cell population dynamics by CSF-1. J Exp Med (2011) 208(9):1901–16. 10.1084/jem.20110866 21825019 PMC3171096

[172] Schreurs MW Eggert AA de Boer AJ Figdor CG Adema GJ. Generation and functional characterization of mouse monocyte-derived dendritic cells. Eur J Immunol (1999) 29(9):2835–41. 10.1002/(SICI)1521-4141(199909)29:09<2835::AID-IMMU2835>3.3.CO;2-H 10508258

[173] Vremec D Lieschke GJ Dunn AR Robb L Metcalf D Shortman K. The influence of granulocyte/macrophage colony-stimulating factor on dendritic cell levels in mouse lymphoid organs. Eur J Immunol (1997) 27(1):40–4. 10.1002/eji.1830270107 9021996

[174] Zhan Y Carrington EM Van Nieuwenhuijze A Bedoui S Seah S Xu Y GM-CSF increases cross-presentation and CD103 expression by mouse CD8^+^ spleen dendritic cells. Eur J Immunol (2011) 41(9):2585–95. 10.1002/eji.201141540 21660938

[175] Sathe P Pooley J Vremec D Mintern J Jin J-O Wu L The acquisition of antigen cross-presentation function by newly formed dendritic cells. J Immunol (2011) 186(9):5184–92. 10.4049/jimmunol.1002683 21422244

[176] Luc S Buza-Vidas N Jacobsen SE. Biological and molecular evidence for existence of lymphoid-primed multipotent progenitors. Ann N Y Acad Sci (2007) 1106:89–94. 10.1196/annals.1392.023 17442777

[177] Verovskaya E Broekhuis MJ Zwart E Ritsema M van Os R de Haan G Heterogeneity of young and aged murine hematopoietic stem cells revealed by quantitative clonal analysis using cellular barcoding. Blood (2013) 122(4):523–32. 10.1182/blood-2013-01-481135 23719303

[178] Paul F Amit I. Plasticity in the transcriptional and epigenetic circuits regulating dendritic cell lineage specification and function. Curr Opin Immunol (2014) 30C:1–8. 10.1016/j.coi.2014.04.004 24820527

[179] Vorhagen S Jackow J Mohor SG Tanghe G Tanrikulu L Skazik-Vogt C Lineage tracing mediated by cre-recombinase activity. J Invest Dermatol (2015) 135(1):e28 10.1038/jid.2014.472 25501382

[180] Srinivas S Watanabe T Lin CS William CM Tanabe Y Jessell TM Cre reporter strains produced by targeted insertion of EYFP and ECFP into the ROSA26 locus. BMC Dev Biol (2001) 1:4. 10.1186/1471-213X-1-4 11299042 PMC31338

[181] Caminschi I Proietto AI Ahmet F Kitsoulis S Shin Teh J Lo JCY The dendritic cell subtype-restricted C-type lectin Clec9A is a target for vaccine enhancement. Blood (2008) 112(8):3264–73. 10.1182/blood-2008-05-155176 18669894 PMC2569177

[182] Sancho D Mourão-Sá D Joffre OP Schulz O Rogers NC Pennington DJ Tumor therapy in mice via antigen targeting to a novel, DC-restricted C-type lectin. J Clin Invest (2008) 118(6):2098–110. 10.1172/JCI34584 18497879 PMC2391066

[183] Liu J Willet SG Bankaitis ED Xu Y Wright CVE Gu G. Non-parallel recombination limits cre-loxP-based reporters as precise indicators of conditional genetic manipulation. Genesis (2013) 51(6):436–42. 10.1002/dvg.22384 23441020 PMC3696028

[184] Nelson PJ Rees AJ Griffin MD Hughes J Kurts C Duffield J. The renal mononuclear phagocytic system. J Am Soc Nephrol (2012) 23(2):194–203. 10.1681/ASN.2011070680 22135312 PMC3269181

[185] Dong X Swaminathan S Bachman LA Croatt AJ Nath KA Griffin MD. Antigen presentation by dendritic cells in renal lymph nodes is linked to systemic and local injury to the kidney. Kidney Int (2005) 68(3):1096–108. 10.1111/j.1523-1755.2005.00502.x 16105040

[186] Hirrlinger J Requardt RP Winkler U Wilhelm F Schulze C Hirrlinger PG. Split-CreERT2: temporal control of DNA recombination mediated by split-Cre protein fragment complementation. PLoS One (2009) 4(12):e8354. 10.1371/journal.pone.0008354 20016782 PMC2791205

[187] Schreiber HA Loschko J Karssemeijer RA Escolano A Meredith MM Mucida D Intestinal monocytes and macrophages are required for T cell polarization in response to *Citrobacter rodentium*. J Exp Med (2013) 210(10):2025–39. 10.1084/jem.20130903 24043764 PMC3782042

[188] Diehl GE Longman RS Zhang J-X Breart B Galan C Cuesta A Microbiota restricts trafficking of bacteria to mesenteric lymph nodes by CX(3)CR1(hi) cells. Nature (2013) 494(7435):116–20. 10.1038/nature11809 23334413 PMC3711636

[189] Ohnmacht C Pullner A King SBS Drexler I Meier S Brocker T Constitutive ablation of dendritic cells breaks self-tolerance of CD4 T cells and results in spontaneous fatal autoimmunity. J Exp Med (2009) 206(3):549–59. 10.1084/jem.20082394 19237601 PMC2699126

[190] Voehringer D Liang H-E Locksley RM. Homeostasis and effector function of lymphopenia-induced “memory-like” T cells in constitutively T cell-depleted mice. J Immunol (2008) 180(7):4742–53. 10.4049/jimmunol.180.7.4742 18354198 PMC2670614

[191] Gautier EL Shay T Miller J Greter M Jakubzick C Ivanov S Gene-expression profiles and transcriptional regulatory pathways that underlie the identity and diversity of mouse tissue macrophages. Nat Immunol (2012) 13(11):1118–28. 10.1038/ni.2419 23023392 PMC3558276

[192] Lavin Y Winter D Blecher-Gonen R David E Keren-Shaul H Merad M Tissue-resident macrophage enhancer landscapes are shaped by the local microenvironment. Cell (2014) 159(6):1312–26. 10.1016/j.cell.2014.11.018 25480296 PMC4437213

[193] Gosselin D Link VM Romanoski CE Fonseca GJ Eichenfield DZ Spann NJ Environment drives selection and function of enhancers controlling tissue-specific macrophage identities. Cell (2014) 159(6):1327–40. 10.1016/j.cell.2014.11.023 25480297 PMC4364385

